# Plasma ascorbic acid and the risk of islet autoimmunity and type 1 diabetes: the TEDDY study

**DOI:** 10.1007/s00125-019-05028-z

**Published:** 2019-11-14

**Authors:** Markus Mattila, Iris Erlund, Hye-Seung Lee, Sari Niinistö, Ulla Uusitalo, Carin Andrén Aronsson, Sandra Hummel, Hemang Parikh, Stephen S. Rich, William Hagopian, Jorma Toppari, Åke Lernmark, Anette G. Ziegler, Marian Rewers, Jeffrey P. Krischer, Jill M. Norris, Suvi M. Virtanen, for the TEDDY Study Group

**Affiliations:** 1 Faculty of Social Sciences/Health Sciences, Tampere University, Tampere, Finland; 2 Department of Public Health Solutions, National Institute for Health and Welfare, PO Box 30, FI-00271 Helsinki, Finland; 3 Department of Government Services, National Institute for Health and Welfare, Helsinki, Finland; 4 Health Informatics Institute, Morsani College of Medicine, University of South Florida, Tampa, FL USA; 5 Department of Clinical Sciences, Lund University, Malmö, Sweden; 6 Institute of Diabetes Research, Helmholtz Zentrum München, Munich, Germany; 7 Forschergruppe Diabetes, Klinikum rechts der Isar, Technische Universität München, Munich, Germany; 8 Forschergruppe Diabetes e.V., Helmhtoltz Zentrum München, Munich, Germany; 9 Center for Public Health Genomics, University of Virginia, Charlottesville, VA USA; 10 Pacific Northwest Research Institute, Seattle, WA USA; 11 Department of Pediatrics, Turku University Hospital, Turku, Finland; 12 Research Centre for Integrative Physiology and Pharmacology, Institute of Biomedicine, University of Turku, Turku, Finland; 13 Barbara Davis Center for Childhood Diabetes, University of Colorado School of Medicine, Aurora, CO USA; 14 Department of Epidemiology, Colorado School of Public Health, University of Colorado Denver, Aurora, CO USA; 15 Center for Child Health Research, Tampere University and Tampere University Hospital, Tampere, Finland; 16 Science Centre, Tampere University Hospital, Tampere, Finland

**Keywords:** Autoimmunity, Plasma ascorbic acid, Single nucleotide polymorphism, SNP, Transporter genes, Type 1 diabetes, Vitamin C

## Abstract

**Aims/hypothesis:**

We studied the association of plasma ascorbic acid with the risk of developing islet autoimmunity and type 1 diabetes and examined whether SNPs in vitamin C transport genes modify these associations. Furthermore, we aimed to determine whether the SNPs themselves are associated with the risk of islet autoimmunity or type 1 diabetes.

**Methods:**

We used a risk set sampled nested case–control design within an ongoing international multicentre observational study: The Environmental Determinants of Diabetes in the Young (TEDDY). The TEDDY study followed children with increased genetic risk from birth to endpoints of islet autoantibodies (350 cases, 974 controls) and type 1 diabetes (102 cases, 282 controls) in six clinical centres. Control participants were matched for family history of type 1 diabetes, clinical centre and sex. Plasma ascorbic acid concentration was measured at ages 6 and 12 months and then annually up to age 6 years. SNPs in vitamin C transport genes were genotyped using the ImmunoChip custom microarray. Comparisons were adjusted for HLA genotypes and for background population stratification.

**Results:**

Childhood plasma ascorbic acid (mean ± SD 10.76 ± 3.54 mg/l in controls) was inversely associated with islet autoimmunity risk (adjusted OR 0.96 [95% CI 0.92, 0.99] per +1 mg/l), particularly islet autoimmunity, starting with insulin autoantibodies (OR 0.94 [95% CI 0.88, 0.99]), but not with type 1 diabetes risk (OR 0.93 [95% Cl 0.86, 1.02]). The *SLC2A2* rs5400 SNP was associated with increased risk of type 1 diabetes (OR 1.77 [95% CI 1.12, 2.80]), independent of plasma ascorbic acid (OR 0.92 [95% CI 0.84, 1.00]).

**Conclusions/interpretation:**

Higher plasma ascorbic acid levels may protect against islet autoimmunity in children genetically at risk for type 1 diabetes. Further studies are warranted to confirm these findings.

**Data availability:**

The datasets generated and analysed during the current study will be made available in the NIDDK Central Repository at https://www.niddkrepository.org/studies/teddy.

**Electronic supplementary material:**

The online version of this article (10.1007/s00125-019-05028-z) contains peer-reviewed but unedited supplementary material, which is available to authorised users.

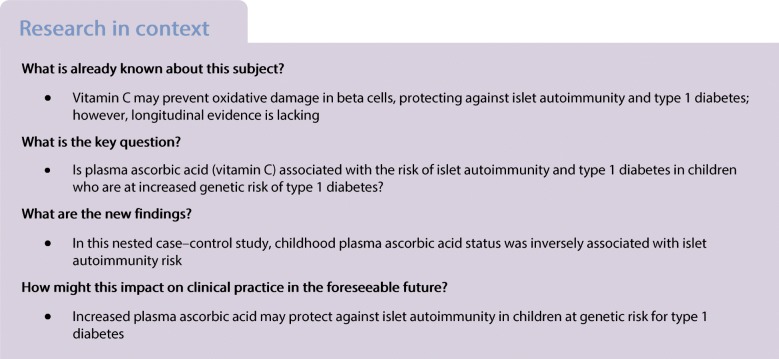



## Introduction

Oxidative stress may play a role in the pathogenesis of type 1 diabetes for several reasons. The cells in pancreatic islets are more vulnerable to oxidative damage than many other cells due to the low activity of free-radical detoxifying and redox-regulating enzymes such as catalase, superoxide dismutase and glutathione peroxidase [1]. It has been hypothesised that dietary antioxidants might improve the islets’ capacity to cope with oxidative stress (e.g. induced by hyperglycaemia) [1–4].

Vitamin C (ascorbic acid) is a water-soluble vitamin obtained from vegetables, fruits and berries [5]. As a dietary antioxidant vitamin C might protect against the development of type 1 diabetes [6]. However, only two case–control studies have investigated the issue. In an Australian study, use of vitamin C supplements was less frequent in children with type 1 diabetes before onset [7]. On the other hand, a Swedish study found no differences in dietary vitamin C intake before onset between type 1 diabetes cases and controls [8]. The association between plasma ascorbic acid concentration and islet autoimmunity or the subsequent development of type 1 diabetes has not been investigated. Plasma ascorbic acid represents the most accurate measure of available vitamin C in the body [9, 10].

Genetic variation in vitamin C metabolic pathways causes inter-individual differences in plasma and tissue ascorbic acid availability [9], which might similarly contribute to type 1 diabetes risk. The metabolism of ascorbic acid is regulated by key proteins called sodium l-ascorbic acid transporters (SVCTs). Two isoforms, hSVCT1 and hSVCT2, encoded by the genes *SLC23A1* and *SLC23A2*, respectively, control the active transport of ascorbic acid across cell membranes and uptake to tissues [11]. hSVCT1 expression is confined to epithelia in renal, intestinal and hepatic tissues, while hSVCT2 is responsible for tissue-specific uptake.

The SNP rs33972313, a low-frequency missense variant in *SLC23A1*, has been consistently associated with lower circulating ascorbic acid status [11]. Other SNPs in *SLC23A1* (intronic SNPs rs6596473 and rs4257763, and promoter SNP rs10063949) have also been associated with ascorbic acid concentration, but less consistently. Furthermore, intronic SNPs (rs6053005 and rs1279683) in *SLC23A2* have been associated with ascorbic acid concentration [11–14]. Less is known about the importance of the second pathway, the solute carrier family 2 (SLC2A; also called GLUT) family proteins that transport dehydroascorbic acid (DHA) into cells where it is converted into ascorbic acid [15].

The aim of this study was to examine the association of plasma ascorbic acid concentration with the risk of islet autoimmunity and type 1 diabetes in children with a high genetic risk of type 1 diabetes. Furthermore, we studied the association of plasma ascorbic acid with the risk of islet autoimmunity relative to the first autoantibody to be observed (either insulin autoantibody [IAA] or GAD autoantibody [GADA], referred to hereafter as IAA first and GADA first, respectively), because the type of autoantibody appearing first may reflect different disease processes [16–18]. In addition, we examined whether vitamin C metabolism genes available on the ImmunoChip are associated with, or modify, the association between plasma ascorbic acid and the development of islet autoimmunity and type 1 diabetes. This included a protein-coding missense SNP in *SLC23A1* (rs33972313), two intronic *SLC2A1* (also known as *GLUT1*, 1p34.2) SNPs (rs1105297 and rs3754223), and an *SLC2A2* (also known as *GLUT2*, 3q26.2) SNP (rs5400) in the dehydro-l-ascorbic acid transport genes.

## Methods

### Study design

The study was performed as a nested case–control study within The Environmental Determinants of the Diabetes in the Young (TEDDY) study. The TEDDY study is an international multicentre observational cohort study that prospectively follows children from birth in the search for environmental factors involved in the development of islet autoimmunity and subsequent type 1 diabetes in genetically susceptible children (based upon HLA genotype), as described in detail previously [19, 20]. Written informed consents have been obtained for all study participants, from a parent or primary caretaker, separately, for genetic screening and participation in prospective follow-up visits. The study is funded by the National Institutes of Health (NIH) and the National Institute of Diabetes and Digestive and Kidney Diseases (NIDDK) and approved by local Institutional Review Boards and monitored by an External Advisory Board. The TEDDY study is conducted in clinical research centres in the USA (Colorado, Georgia/Florida, Washington State), Finland, Sweden and Germany.

### Study population

Between 1 September 2004 and 28 February 2010, a total of 424,788 new-born infants were screened for type 1 diabetes-associated HLA genotypes. Eligibility criteria for initial contact was one of the following HLA class II genotypes: *HLA-DR3/4*; *HLA-DR4/4*; *HLA-DR4/8*; *HLA-DR3/3* and *HLA*-*DR4/4*. Infants with HLA-DR genotypes *HLA-DR4/1*, *HLA-DR4/13*, *HLA-DR4/9* and *HLA-DR3/9* were included only if they had a first-degree relative (i.e. mother, father or sibling) with type 1 diabetes [21]. Of the eligible infants, 21,589 had type 1 diabetes genetic risk based on HLA genotyping and 8676 children were enrolled. Children were followed every 3 months with a scheduled clinic visit until the age of 4 years, and every 6 months thereafter until being diagnosed with type 1 diabetes. Children with islet autoimmunity were followed every 3 months throughout the study. Of the enrolled children, 2211 (25.5% of the 8676) were withdrawn or lost to follow-up by 6 years of age at the time of the design.

### Outcomes

The primary outcomes in this study were persistent confirmed islet autoimmunity and the diagnosis of type 1 diabetes. Persistent confirmed islet autoimmunity was defined by appearance of one or more islet cell autoantibodies (IAA, GAD, or autoantibody to tyrosine phosphatase-related islet antigen 2 [IA-2A], also known as insulinoma antigen-2 antibody) confirmed at two consecutive visits. Type 1 diabetes diagnosis was based on American Diabetes Association criteria [22]. Different autoantibodies may be associated with different disease processes [16–18] and therefore secondary analyses were conducted regarding timing of autoantibodies: IAA first and GADA first.

### Nested case–control design

The current study was performed with risk set sampling described previously [23]. The study was conducted in two nested case–control designs within the TEDDY study: (1) for islet autoimmunity outcome; and (2) for type 1 diabetes outcome. Three matched controls were selected per islet autoimmunity and type 1 diabetes case. Children were matched for family history of type 1 diabetes, clinical centre and sex. The nested case–control study sets were based on the data collected as of 31 May 2012 [23].

The islet autoimmunity outcome analysis included 350 cases with median seroconversion age of 23 months (range 6–72 months) and 974 matched controls. Islet autoimmunity cases were defined as a participant with persistent islet autoimmunity. A control was defined as a participant who had not developed persistent islet autoimmunity by the time that the corresponding matched case had done, plus 45 days. The islet autoimmunity case–control set used for the statistical analyses consisted of 3371 plasma samples taken for ascorbic acid measurement. Three hundred and sixty-five samples failed the laboratory’s internal quality control. Samples from controls were processed only when the matched case had an available sample at a corresponding visit. The mean ascorbic acid level for each child was calculated from all available measurements.

Type 1 diabetes outcome analysis consisted of 102 cases with median age of 31 months at diagnosis (range 8–75 months) and 282 matched controls. A control for a type 1 diabetes case was defined as a participant who had not developed type 1 diabetes by the time the corresponding matched case had done so, plus 45 days. Of the 350 islet autoimmunity cases, 74 were also analysed as type 1 diabetes cases. Those islet autoimmunity cases who developed type 1 diabetes but had seroconverted already before the first plasma sample was collected for ascorbic acid measurement (collection started at 6 months of age) were included only in the type 1 diabetes analysis. Thus, 28 type 1 diabetes cases were included only for type 1 diabetes analysis.

Secondary outcome analyses were performed within the islet autoimmunity nested case–control design. The islet autoimmunity dataset included 163 IAA first cases with median seroconversion age of 18 months (range 6–72 months) and 450 controls. Within the islet autoimmunity dataset there were 120 GADA first cases with median seroconversion age of 28 months (range 6–68 months) with 336 controls.

#### Plasma ascorbic acid measurements

Plasma samples for ascorbic acid measurement were collected at the age of 6 months and 12 months, and then annually until 6 years of age or until and including the time of seroconversion of the islet autoimmunity cases, and for type 1 diabetes cases the visit just preceding the type 1 diabetes diagnosis (with corresponding time for matched controls). Stabilisers were added to plasma samples intended for ascorbic acid analysis before freezing to minimise degradation of ascorbic acid [24]. After sample collection at the clinical centres, 50 μl of sodium citrate plasma (in BD Vacutainer CPT Cell Preparation Tubes [Becton Dickinson, Franklin Lakes, NJ, USA]) was transferred into cryovials and 0.2 ml of 5% (wt/vol.) trichloroacetic acid and 200 mg disodium EDTA was added, with subsequent freezing at −70°C. A long-distance protocol was developed for the collection of blood samples from families living away from their nearest TEDDY study clinic (most frequently being the case in Germany compared with the other countries). In the long-distance collection protocol, blood samples were obtained by a family paediatrician and transported within 24 h to the TEDDY study clinic site. Case samples were paired with matched control samples and randomly placed within a batch before samples were transported to the laboratory.

Ascorbic acid measurements were performed at the Biochemistry Laboratory, Genomics and Biomarkers Unit, National Institute for Health and Welfare (THL), Helsinki, Finland. Ascorbic acid was determined by an ion-paired, reversed-phase, high-performance liquid chromatographic method using electrochemical detection, as described [24]. Isoascorbic acid was used as internal standard for the quantification of ascorbic acid. The laboratory staff were blinded to the case–control status of samples. The laboratory included three internal quality control samples of three ascorbic acid levels in each run (altogether nine samples). Precision within the project, expressed as the CV of the quality control samples, was 9.2–12.6% at a concentration range of 4.6–11.2 mg/l. During the project, the laboratory participated three times in an external quality assessment scheme (National Institute of Standards and Technology (NIST) Micronutrients Measurement Quality Assurance Program for Total Ascorbic Acid). The results were in excellent concordance with NIST values.

#### Genotyping

Illumina Infinium ImmunoChip custom microarray was used for SNP genotyping, based upon robust genome-wide association analyses (GWAS) in 12 autoimmune diseases (including type 1 diabetes). The ImmunoChip array contained 195,806 SNPs that were genotyped on TEDDY study DNA samples. Principal components analysis (PCA) using EIGENSTRAT software (Department of Genetics, Harvard Medical School, Boston, MA, USA) was performed using each unrelated TEDDY study participant to estimate ancestry, with the two most significant principal components used as covariates in analytical models. For our primary hypothesis on SNPs in ascorbic acid pathways, *SLC23A1* (rs33972313), *SLC2A1* (rs1105297 and rs3754223) and *SLC2A2* (rs5400) were on the ImmunoChip, passed quality control metrics and were selected for analysis.

#### Statistical analyses

A linear mixed effects model adjusted for the case–control status was used to examine whether plasma ascorbic acid over time was different by the use of standard vs long-distance protocol, country, SNPs and other risk factors for type 1 diabetes. The random effects for participant were nested within the random effects for case–control set in the model. Weight *z* score and height *z* score were derived from Centers for Disease Control and Prevention standardised growth charts.

Plasma ascorbic acid was analysed up to the case’s event age specific to each nested case–control set. The measures were from all visits prior to and including the first of two consecutive visits at which the child tested positive for an autoantibody for islet autoimmunity analysis and prior to the diagnosis for type 1 diabetes analysis. The mean of the corresponding measures for each participant (childhood ascorbic acid) was compared between matched case and control children. Conditional logistic regression was used to assess the association between the characteristics of interest and the case–control status. Interaction term with the matching factors was tested for the effect modification. All analyses were adjusted for HLA genotype (*HLA-DR3/4* vs other) and two principal components of ancestry to control for population stratification. The log-linearity of the characteristic with each outcome was examined using the supremum test [25]. All analyses were performed using SAS 9.4 (SAS Institute, Cary, NC, USA). A two-sided *p* value <0.05 was considered statistically significant.

## Results

### Background characteristics

The mean and SD of childhood plasma ascorbic acid concentrations are shown according to matching variables in islet autoimmunity and type 1 diabetes case–control children in Table 1. The mean (± SD) plasma ascorbic acid concentration for islet autoimmunity cases and controls was 10.21 ± 3.33 mg/l and 10.76 ± 3.54 mg/l, respectively. For the type 1 diabetes cases and controls, the mean (± SD) plasma ascorbic acid concentration was 9.73 ± 3.18 mg/l and 10.58 ± 3.57 mg/l, respectively.

**Table 1:** Mean childhood plasma ascorbic acid in islet autoimmunity and type 1 diabetes cases and controls

Matching variable	Islet autoimmunity			Type 1 diabetes		
	No. (%) of cases	Plasma ascorbic acid concentration (mg/l)^a^		No. (%) of cases	Plasma ascorbic acid concentration (mg/l)^a^	
		Cases	Controls		Cases	Controls
Clinical centre						
Colorado	51 (14.6)	11.7 ± 3.0	12.2 ± 3.3	15 (14.7)	12.0 ± 2.5	12.2 ± 3.1
Georgia	24 (6.9)	12.5 ± 3.5	12.3 ± 3.4	6 (5.9)	13.1 ± 4.2	13.6 ± 4.1
Washington State	34 (9.7)	11.4 ± 4.3	11.8 ± 4.4	7 (6.9)	9.8 ± 3.5	11.6 ± 3.2
Finland	105 (30.0)	10.5 ± 2.8	10.7 ± 3.0	35 (34.3)	9.8 ± 2.7	10.7 ± 3.6
Germany	26 (7.4)	9.2 ± 2.5	9.7 ± 3.4	15 (14.7)	10.2 ± 3.2	10.3 ± 3.8
Sweden	110 (31.4)	8.7 ± 3.0	9.5 ± 3.6	24 (23.5)	7.9 ± .9	8.8 ± 3.0
Sex						
Female	157 (44.9)	10.0 ± 3.4	10.7 ± 3.5	47 (46.1)	10.2 ± 3.1	10.2 ± 3.1
Male	193 (55.1)	10.4 ± 3.2	10.8 ± 3.7	55 (53.9)	9.7 ± 3.3	11.2 ± 4.0
FDR/GP status						
FDR	76 (21.7)	10.8 ± 3.1	11.1 ± 3.7	36 (35.3)	10.8 ± 2.8	11.0 ± 3.8
GP	274 (78.3)	10.0 ± 3.4	10.7 ± 3.5	66 (64.7)	9.5 ± 3.4	10.6 ± 3.6

Plasma ascorbic acid concentrations are presented as mean ± SD

^a^Mean childhood plasma ascorbic acid; includes measures from all visits prior to and including the seroconversion visit, which is the first of two consecutive visits at which the child tested positive for an autoantibody. To convert ascorbic acid concentration to μmol/l, multiply values in mg/l by 5.678

FDR, first-degree relative of an individual with type 1 diabetes; GP, from the general population (no first-degree relative with type 1 diabetes)

Plasma ascorbic acid concentrations did not differ by the use of standard protocol vs long-distance protocol. As potential risk factors for type 1 diabetes, we examined growth variables (mean height and weight *z* score prior to the outcome), as well as breastfeeding status at 3 and 6 months. Breastfeeding was associated with lower plasma ascorbic acid status (mixed model regression variables estimate [SE]: −0.96 [0.26], *p* < 0.001 for breastfeeding vs not breastfeeding at 3 months and −0.77 [0.23], *p* < 0.001 for breastfeeding vs not breastfeeding at 6 months) but no significant association was found with the outcomes. Higher weight or height *z* scores were associated with lower plasma ascorbic acid concentration (−0.28 [0.10], *p* = 0.006 and −0.12 [0.04], *p* = 0.001, respectively). Because weight was also associated with islet autoimmunity (OR [95% CI]: 1.23 [1.07, 1.41] for any islet autoimmunity; 1.24 [1.01, 1.51] for IAA first; 1.32 [1.05, 1.65] for GADA first), we adjusted for mean weight *z* score in the models examining ascorbic acid concentration vs outcomes.

### Primary outcomes: islet autoimmunity and type 1 diabetes

Childhood plasma ascorbic acid status was inversely associated with islet autoimmunity (OR 0.96 [95% CI 0.92, 0.99], *p* = 0.04) but the association with type 1 diabetes risk was not significant (OR 0.93 [95% CI 0.86 1.02], *p* = 0.11) (Table 2). Adjustment for mean weight *z* score prior to the outcome showed a similar pattern: islet autoimmunity OR 0.96 (95% CI 0.92, 1.00), *p* = 0.06; type 1 diabetes OR 0.93 (95% CI 0.86, 1.02), *p* = 0.11. All the outcome analyses were adjusted for ethnicity and *HLA-DR3/4* genotype. The association between plasma ascorbic acid concentration and risk of islet autoimmunity and type 1 diabetes was not modified by the participant *HLA-DR3/4* genotype, clinical centre, sex or family history of type 1 diabetes.

**Table 2:** Risk of type 1 diabetes-related outcomes associated with childhood plasma ascorbic acid

Outcome	OR (95% CI)^a^	*p* value
Islet autoimmunity (cases, *n* = 350)	0.96 (0.92, 0.99)	0.041
Type 1 diabetes (cases, *n* = 102)	0.93 (0.86, 1.02)	0.109
IAA first (cases, *n* = 163)	0.94 (0.88, 0.99)	0.028
GADA first (cases, *n* = 120)	0.99 (0.93, 1.07)	0.988

Data are presented as OR (95% CI) per 1 mg/l increase in childhood ascorbic acid concentration

Mean childhood plasma ascorbic acid includes measures from all visits prior to and including the seroconversion visit, which is the first of two consecutive visits at which the child tested positive for an autoantibody, and for type 1 diabetes all visits prior to diagnosis. To convert ascorbic acid concentration to μmol/l, multiply values in mg/l by 5.678

^a^Adjusted for two largest principal components for ethnicity and *HLA-DR3/4* genotype

### Secondary outcomes: IAA first and GADA first

Childhood plasma ascorbic acid status was inversely associated with the risk of IAA first (OR 0.94 [95% CI 0.88, 0.99], *p* = 0.03). Adjustment for mean weight *z* score prior to the outcome produced similar results (OR 0.93 [95% CI 0.88, 0.99], *p* = 0.03). Plasma ascorbic acid concentration was not associated with GADA first (OR 0.99 [95% CI 0.93, 1.07], *p* = 0.99). All the outcome analyses were adjusted for ethnicity and *HLA-DR3/4* genotype. The *HLA-DR3/4* genotype did not modify the association between plasma ascorbic acid concentration and the risk of IAA first or risk of GADA first.

### SNPs and outcomes and effect modification by SNPs

The *SLC23A1* rs33972313 minor allele carriers had lower mean plasma ascorbic acid concentration than non-carriers (mixed model regression variable estimate [SE]: −2.22 [0.46], *p* < 0.001). However, the SNP was not associated with the risk of islet autoimmunity or type 1 diabetes (Table 3), nor with IAA first or GADA first (data not shown). Furthermore, *SLC23A1* rs33972313 did not modify the association between plasma ascorbic acid and the risk of islet autoimmunity or type 1 diabetes (Table 3), IAA first (interaction *p* = 0.43) or GADA first (interaction *p* = 0.10). DHA transport gene SNPs in *SLC2A1* (rs1105297 and rs3754223) were not associated with plasma ascorbic acid concentration (*p* = 0.32 and *p* = 0.76) or the risk of islet autoimmunity, type 1 diabetes, IAA first or GADA first, either alone or in conjunction with the plasma ascorbate status. They did not modify the association between plasma ascorbic acid and the risk of islet autoimmunity or type 1 diabetes (Table 3), IAA first or GADA first (data not shown).

**Table 3:** Risk of islet autoimmunity and type 1 diabetes associated with ascorbic acid transport gene polymorphisms and effect modification between the genes and childhood plasma ascorbic acid on the islet autoimmunity and type 1 diabetes risk

Gene	SNP (minor allele)	Islet autoimmunity				Type 1 diabetes			
		% of minor allele, cases/controls	OR (95% CI)^a^	*p* value^a^	*p* value^b^	% of minor allele, cases /controls	OR (95% CI)^a^	*p* value^a^	*p* value^b^
*SLC23A1*^c^	rs33972313 (A)	3.4/2.8	1.18 (0.70, 1.99)	0.533	0.158	5.4/1.6	2.52 (0.96, 6.59)	0.060	0.101
*SLC2A1*^c^	rs1105297 (A)	33.7/32.8	1.04 (0.86, 1.26)	0.690	0.094	33.8/32.6	1.09 (0.75, 1.56)	0.661	0.175
*SLC2A1*^c^	rs3754223 (A)	21.9/22.7	0.92 (0.74, 1.15)	0.473	0.959	25.5/21.6	1.40 (0.93, 2.11)	0.107	0.665
*SLC2A2*^c^	rs5400 (A)	12.7/13.7	0.90 (0.69, 1.16)	0.408	0.456	18.6/11.7	1.66 (1.06, 2.60)	0.028	0.785

^a^Adjusted for two largest principal components for ethnicity and *HLA-DR3/4* genotype

^b^Indication of interaction of childhood plasma ascorbic acid with the number of transport gene SNP alleles on the risk of islet autoimmunity and type 1 diabetes, adjusted for two largest principal components for ethnicity and *HLA-DR3/4* genotype

^c^Genetic data were missing from two islet autoimmunity cases, one type 1 diabetes case and five controls

*SLC2A2* rs5400 was not associated with plasma ascorbic acid concentration (*p* = 0.54) or the risk of islet autoimmunity, IAA first or GADA first; however, *SLC2A2* rs5400 was associated with increased risk of type 1 diabetes (OR 1.66 [95% CI 1.06, 2.60], *p* = 0.028) (Table 3). The association remained even after adjusting for plasma ascorbic acid in addition to the two largest principal components for ethnicity and *HLA-DR3/4* genotype. In this model, rs5400 was associated with increased risk of type 1 diabetes (OR 1.77 [95% CI 1.12, 2.80], *p* = 0.015), while plasma ascorbic acid concentration showed no association (OR 0.92 [95% CI 0.84, 1.00], *p* = 0.058). The *SLC2A2* rs5400 SNP did not modify the association of plasma ascorbic acid status with the risk of islet autoimmunity or type 1 diabetes, IAA first or GADA first.

## Discussion

In this relatively large prospective study, mean ascorbic acid concentration in plasma was inversely associated with the risk of islet autoimmunity, but not type 1 diabetes, in children with increased genetic risk of type 1 diabetes. The association between ascorbic acid and type 1 diabetes was, however, only marginally different and showed a stronger point estimate compared with islet autoimmunity. The SNPs investigated in our study (i.e. SNPs in ascorbic acid or dehydroascorbic acid transport genes) did not modify the observed associations with islet autoimmunity and the SNPs themselves were not associated with islet autoimmunity. However, the presence of the minor alleles in *SLC2A2* rs5400 appeared to increase the risk of type 1 diabetes. Furthermore, SNP rs33972313 in *SLC23A1* was associated with lower plasma ascorbic acid concentrations, in line with previous studies, and appeared to increase type 1 diabetes risk, although the finding was of borderline significance (*p* = 0.06).

One of the strengths of our study is the large multinational study sample with multiple measurements of plasma ascorbic acid as well as genetic information. To our best knowledge, our study is the first one to assess prospectively whether plasma ascorbic acid concentration (and genetic variation related to ascorbic acid) is associated with development of islet autoimmunity and type 1 diabetes. Measurement of ascorbic acid concentration in the plasma may more accurately reflect availability to tissues, as compared with dietary intake measurements. Previous case–control studies have assessed ascorbic acid intake from diet and supplements using questionnaire [7, 8]. Another strength is that we were able to investigate the endpoints of IAA first and GADA first separately; this is important because they may reflect different disease processes. IAA usually appears during the first to second year of life, whereas GADA usually appears at 3–5 years of age or even later [26, 27]. In other words, we were able to study associations of plasma ascorbic acid at very early stages of autoimmunity development. A limitation of our study is the use of array platform for measuring genetic information on SNPs, as this might not determine the target genes accurately. Furthermore, besides HLA type and ethnicity, we could not take into account other potential confounding factors that might affect ascorbic acid status (e.g. endogenous stress such as infection or genetic variation of proteins regulating oxidative stress such as glutathione *S*-transferases and haptoglobin [9, 10]). Other limitations of our study are that it included only children who developed type 1 diabetes at a very early age (mean age of diagnosis 29 months) and that the follow-up time (up to 6 years of age) for type 1 diabetes was relatively short.

Our prospective study shows that higher plasma ascorbic acid concentration was associated with lower risk of islet autoimmunity, in particular with lower risk of IAA first. The results indicate that vitamin C might be protective, particularly during the early stages of autoimmunity development or early in life. This is in line with previous findings linking other early dietary exposures to IAA [16, 28].

Our study confirms previous findings that carrying the *SLC23A1* gene SNP rs33972313 minor alleles results in reduced plasma ascorbic acid concentration [11]. The presence of the minor ‘A’ allele, results in a change from the valine (Val/GTG) form to the methionine (Meth/ATG) form [11]. This alters the function the vitamin C transport proteins, impairing the active transport of ascorbic acid via decreased renal reabsorption, increased excretion and altered dose–response of plasma ascorbic acid [29].

Our study included SNPs in genes involved in the transport of DHA as well as ascorbic acid. Genetic variation in ascorbic acid transport would appear more important, because vitamin C mainly enters cells as l-ascorbic acid. However, vitamin C is also taken up as DHA, which is the oxidised form of ascorbic acid and is reduced back to ascorbic acid intracellularly. DHA transport occurs through five glucose transporters encoded by the *SLC2A* solute carrier gene family [30–33]. SNPs in any of these transport-protein-encoding genes could affect cellular ascorbate status and they are therefore all of interest in our study.

None of the SNPs investigated modified the associations between plasma ascorbic acid and the outcomes. In addition, our results do not imply any association between the SNPs and islet autoimmunity. However, a common variant in the *SLC2A2* gene (rs5400) was associated with an increased risk of type 1 diabetes, while a less frequent variant in *SLC23A1* (rs33972313) showed an association of borderline statistical significance. More studies are needed to corroborate the findings and to investigate the underlying mechanisms, because they may be related to other functions of these proteins.

Of the SNPs investigated, only rs33972313 in the *SLC23A1* gene has been shown to reduce ascorbic acid status [11]. SLC2A proteins are well-known glucose transporters, with the proteins encoded by *SLC2A1* and *SLC2A3* being more essential for pancreatic glucose transport in humans, compared with proteins encoded by *SLC2A2*, because they are expressed at higher levels. A previous study found an association between *SLC2A2* SNP rs5400 and an increased risk of type 2 diabetes [34] but we are not aware of studies linking the SNP to type 1 diabetes.

In this study, children who were not breastfed at 3 or 6 months of age had higher plasma ascorbic acid concentration compared with children who were breastfed for longer. This may result from early introduction of complementary foods containing higher amounts of vitamin C. Although a child’s plasma ascorbic acid status might be affected by maternal intake of vitamin C, there are no studies assessing the association between maternal intake of vitamin C during lactation and a child’s risk of islet autoimmunity and type 1 diabetes. The mother’s adequate vitamin C status during pregnancy is important to the development of the fetus [35] and may affect the child’s risk of developing type 1 diabetes. However, in a previous study, maternal vitamin C intake during pregnancy was found not to be associated with the child’s risk of islet autoimmunity or type 1 diabetes in a population with adequate vitamin C intake [36]. It should be noted that the associations between ascorbic acid and type 1 diabetes outcomes may be different at different intake levels or plasma concentrations and inter-individual differences could also play a role. Different results may be observed in studies performed in vitamin C-deficient populations or in supplementation trials.

It has been suggested that higher weight gain during infancy and/or childhood is related to increased risk of islet autoimmunity and type 1 diabetes [37, 38]. In the current study, mean weight prior to islet autoimmunity was associated with islet autoimmunity outcomes but adjustment for weight did not change the association between plasma ascorbic acid concentration and the outcomes.

The associations observed in this study are novel and relatively weak. Further studies are therefore needed to corroborate the findings.

### Conclusions

Higher plasma ascorbic acid may reduce the risk of islet autoimmunity in children with increased genetic risk of type 1 diabetes. Furthermore, genetic variation in vitamin C and glucose transporters might play a role in the development of type 1 diabetes. Further studies are warranted to elucidate the role of vitamin C in type 1 diabetes development.

## Electronic supplementary material

ESM 1 (PDF 203 kb)

## Data Availability

The datasets generated and analysed during the current study will be made available in the NIDDK Central Repository at https://www.niddkrepository.org/studies/teddy.

## Abbreviations

DHA: Dehydroascorbic acid
GADA: GAD autoantibody
IAA: Insulin autoantibody
IA-2A: Autoantibody to tyrosine phosphatase-related islet antigen 2
NIST: National Institute of Standards and Technology
SLC2A: Solute carrier family 2
SVCT: Sodium l-ascorbic acid transporter
TEDDY: The Environmental Determinants of Diabetes in the Young
