# Supplementary material for: Plasma ascorbic acid and the risk of islet autoimmunity and type 1 diabetes: the TEDDY study
Source: Diabetologia. 2019 Nov 14;63(2):278–86. doi: 10.1007/s00125-019-05028-z (PMC6946743; doi:10.1007/s00125-019-05028-z)
Supplement: Supplementary file 1 — (PDF 203 kb) [file 125_2019_5028_MOESM1_ESM.pdf]

Supplementary material: The TEDDY Study Group

Colorado Clinical Center: Marian Rewers, M.D., Ph.D., PI[1,4,5,6,9,10], Aaron Barbour, Kimberly Bautista[11], Judith Baxter[8,911], Daniel Felipe-Morales, Kimberly Driscoll, Ph.D.[8], Brigitte I. Frohnert, M.D.[2,13], Marisa Stahl, M.D.[12], Patricia Gesualdo[2,6,11,13], Michelle Hoffman[11,12,13], Rachel Karban[11], Edwin Liu, M.D.[12], Jill Norris, Ph.D.[2,3,11], Stesha Peacock, Hanan Shorrosh, Andrea Steck, M.D.[3,13], Megan Stern, Erica Villegas[2], Kathleen Waugh[6,7,11]. University of Colorado, Anschutz Medical Campus, Barbara Davis Center for Childhood Diabetes.

Finland Clinical Center: Jorma Toppari, M.D., Ph.D., PI[¥^1,4,10,13], Olli G. Simell, M.D., Ph.D., Annika Adamsson, Ph.D.[^11], Suvi Ahonen[*±§], Mari Åkerlund[*±§], Leena Hakola[*], Anne Hekkala, M.D.[µ¤], Henna Holappa[µ¤], Heikki Hyöty, M.D., Ph.D.[*±6], Anni Ikonen[µ¤], Jorma Ilonen, M.D., Ph.D.[¥¶3], Sinikka Jäminki[*±], Sanna Jokipuu[^], Leena Karlsson[^], Jukka Kero M.D., Ph.D.[¥^], Miia Kähönen[µ¤11,13], Mikael Knip, M.D., Ph.D.[*±5], Minna-Liisa Koivikko[µ¤], Merja Koskinen[*±], Mirva Koreasalo[*±§2], Kalle Kurppa, M.D., Ph.D.[*±12], Jarita Kytölä[*±], Tiina Latva-aho[µ¤], Katri Lindfors, Ph.D.[*12], Maria Lönnrot, M.D., Ph.D.[*±6], Elina Mäntymäki[^], Markus Mattila[*], Maija Miettinen[§2], Katja Multasuo[µ¤], Teija Mykkänen[µ¤], Tiina Niininen[±*11], Sari Niinistö[±§2], Mia Nyblom[*±], Sami Oikarinen, Ph.D.[*±], Paula Ollikainen[µ¤], Zhian Othmani[^], Sirpa Pohjola[µ¤], Petra Rajala[^], Jenna Rautanen[±§], Anne Riikonen[*±§2], Eija Riski[^], Miia Pekkola[*±], Minna Romo[^], Satu Ruohonen[^], Satu Simell, M.D., Ph.D.[¥12], Maija Sjöberg[^], Aino Stenius[µ¤11], Päivi Tossavainen, M.D.[µ¤], Mari Vähä-Mäkilä[¥], Sini Vainionpää[^], Eeva Varjonen[^11], Riitta Veijola, M.D., Ph.D.[µ¤13], Irene Viinikangas[µ¤], Suvi M. Virtanen, M.D., Ph.D.[*±§2]. [¥]University of Turku, *Tampere University, [µ]University of Oulu, [^]Turku University Hospital, Hospital District of Southwest Finland, [±]Tampere University Hospital, [¤]Oulu University Hospital, §National Institute for Health and Welfare, Finland, [¶]University of Eastern Finland.

Georgia/Florida Clinical Center: Jin-Xiong She, Ph.D., PI[1,3,4,10], Desmond Schatz, M.D.[*4,5,7,8], Diane Hopkins[11], Leigh Steed[11,12,13], Jennifer Bryant[11], Katherine Silvis[2], Michael Haller, M.D.[*13],

Melissa Gardiner[11], Richard McIndoe, Ph.D., Ashok Sharma, Stephen W. Anderson, M.D.^, Laura Jacobsen, M.D.*[13], John Marks, DHSc.*[11,13], P.D. Towe*. Center for Biotechnology and Genomic Medicine, Augusta University. *University of Florida, ^Pediatric Endocrine Associates, Atlanta.

Germany Clinical Center: Anette G. Ziegler, M.D., PI[1,3,4,10], Ezio Bonifacio Ph.D.*[5], Anita Gavrisan, Cigdem Gezginci, Anja Heublein, Verena Hoffmann, Ph.D.[2], Sandra Hummel, Ph.D.[2], Andrea Keimer¥[2], Annette Knopff[7], Charlotte Koch, Sibylle Koletzko, M.D.¶[12], Claudia Ramminger[11], Roswith Roth, Ph.D.[8], Marlon Scholz, Joanna Stock[8,11,13], Katharina Warncke, M.D.[13], Lorena Wendel, Christiane Winkler, Ph.D.[2,11]. Forschergruppe Diabetes e.V. and Institute of Diabetes Research, Helmholtz Zentrum München, Forschergruppe Diabetes, and Klinikum rechts der Isar, Technische Universität München. *Center for Regenerative Therapies, TU Dresden, ¶Dr. von Hauner Children's Hospital, Department of Gastroenterology, Ludwig Maximillians University Munich, ¥University of Bonn, Department of Nutritional Epidemiology.

Sweden Clinical Center: Åke Lernmark, Ph.D., PI[1,3,4,5,6,8,9,10], Daniel Agardh, M.D., Ph.D.[6,12], Carin Andrén Aronsson, Ph.D.[2,11,12], Maria Ask, Rasmus Bennet, Corrado Cilio, Ph.D., M.D.[5,6], Helene Engqvist, Emelie Ericson-Hallström, Annika Fors, Lina Fransson, Thomas Gard, Monika Hansen, Hanna Jisser, Fredrik Johansen, Berglind Jonsdottir, M.D., Ph.D.[11], Silvija Jovic, Helena Elding Larsson, M.D., Ph.D.[6,13], Marielle Lindström, Markus Lundgren, M.D., Ph.D.[13], Marlena Maziarz, Ph.D., Maria Månsson-Martinez, Maria Markan, Jessica Melin[11], Zeliha Mestan, Caroline Nilsson, Karin Ottosson, Kobra Rahmati, Anita Ramelius, Falastin Salami, Anette Sjöberg, Birgitta Sjöberg, Malin Svensson, Carina Törn, Ph.D.[3], Anne Wallin, Åsa Wimar[13], Sofie Åberg. Lund University.

Washington Clinical Center: William A. Hagopian, M.D., Ph.D., PI[1,3,4,5,6,7,10,12,13], Michael Killian[6,7,11,12], Claire Cowen Crouch[11,13], Jennifer Skidmore[2], Masumeh Chavoshi, Rachel Hervey, Rachel Lyons, Arlene Meyer, Denise Mulenga[11], Jared Radtke, Matei Romancik, Davey Schmitt, Sarah Zink. Pacific Northwest Research Institute.

Pennsylvania Satellite Center: Dorothy Becker, M.D., Margaret Franciscus, MaryEllen Dalmagro-Elias Smith[2], Ashi Daftary, M.D., Mary Beth Klein, Chrystal Yates. Children's Hospital of Pittsburgh of UPMC.

Data Coordinating Center: Jeffrey P. Krischer, Ph.D.,PI[1,4,5,9,10], Sarah Austin-Gonzalez, Maryouri Avendano, Sandra Baethke, Rasheedah Brown[11], Brant Burkhardt, Ph.D.[5,6], Martha Butterworth[2], Joanna Clasen, David Cuthbertson, Stephen Dankyi, Christopher Eberhard, Steven Fiske[8], Jennifer Garmeson, Veena Gowda, Kathleen Heyman, Belinda Hsiao, Christina Karges, Francisco Perez Laras, Hye-Seung Lee, Ph.D.[1,2,3,12], Qian Li[2,3], Shu Liu, Xiang Liu, Ph.D.[2,3813], Kristian Lynch, Ph.D. [5,6,8], Colleen Maguire, Jamie Malloy, Cristina McCarthy[11], Aubrie Merrell, Hemang Parikh, Ph.D.[3], Ryan Quigley, Cassandra Remedios, Chris Shaffer, Laura Smith, Ph.D.[8,11], Susan Smith[11], Noah Sulman, Ph.D., Roy Tamura, Ph.D.[1,2,11,12,13], Dena Tewey, Michael Toth, Ulla Uusitalo, Ph.D.[2], Kendra Vehik, Ph.D.[4,5,6,8,13], Ponni Vijayakandipan, Keith Wood, Jimin Yang, Ph.D., R.D.[2]. *Past staff: Michael Abbondondolo, Lori Ballard, David Hadley, Ph.D., Wendy McLeod, Steven Meulemans.* University of South Florida.

Project scientist: Beena Akolkar, Ph.D.[1,3,4,5,6,7,9,10]. National Institutes of Diabetes and Digestive and Kidney Diseases.

Autoantibody Reference Laboratories: Liping Yu, M.D.[^5], Dongmei Miao, M.D.[^], Polly Bingley, M.D., FRCP*[5], Alistair Williams*, Kyla Chandler*, Olivia Ball*, Ilana Kelland*, Sian Grace*. [^]Barbara Davis Center for Childhood Diabetes, University of Colorado Denver, *Bristol Medical School, University of Bristol, UK.

Dietary Biomarkers Laboratory: Iris Erlund, Ph.D.[2], Irma Salminen, Jouko Sundvall, Nina Kangas, Petra Arohonka. National Institute for Health and Welfare, Helsinki, Finland.

HLA Reference Laboratory: William Hagopian[3], MD, PhD, Masumeh Chavoshi, Jared Radtke, Sarah Zink. Pacific Northwest Research Institute, Seattle WA. (Previously Henry Erlich, Ph.D.[3],

Steven J. Mack, Ph.D., Anna Lisa Fear. Center for Genetics, Children's Hospital Oakland Research Institute.)

SNP Laboratory: Stephen S. Rich, Ph.D.[3], Wei-Min Chen, Ph.D.[3], Suna Onengut-Gumuscu, Ph.D.[3], Emily Farber, Rebecca Roche Pickin, Ph.D., Jonathan Davis, Jordan Davis, Dan Gallo, Jessica Bonnie, Paul Campolieto. Center for Public Health Genomics, University of Virginia.

Repository: Sandra Ke, Niveen Mulholland, Ph.D. NIDDK Biosample Repository at Fisher BioServices.

Other contributors: Kasia Bourcier, Ph.D.[5], National Institutes of Allergy and Infectious Diseases. Thomas Briese, Ph.D.[6], Columbia University. Suzanne Bennett Johnson, Ph.D.[8,11], Florida State University. Eric Triplett, Ph.D.[6], University of Florida.

Committees:

[1]Ancillary Studies, [2]Diet, [3]Genetics, [4]Human Subjects/Publicity/Publications, [5]Immune Markers, [6]Infectious Agents, [7]Laboratory Implementation, [8]Psychosocial, [9]Quality Assurance, [10]Steering, [11]Study Coordinators, [12]Celiac Disease, [13]Clinical Implementation.
